# Willingness of youth without disabilities to have romantic love and marital relationships with persons with disabilities

**DOI:** 10.1186/s40504-021-00114-w

**Published:** 2021-06-21

**Authors:** Bewunetu Zewude, Tewodros Habtegiorgis

**Affiliations:** Department of Sociology, Wolaita Sodo University, Sodo, Ethiopia

**Keywords:** Disability, Relationship, Sexuality, Stereotype, Youth without disability

## Abstract

People with disabilities face attitudinal barriers including prejudice, stereotypes, and low expectations. Many young people without disabilities may doubt that people with disabilities can be fulfilling partners in any loving adult relationship. The objective of the present research was to assess the willingness of non-disabled youth to engage in conjugal relationships with persons with disabilities in Wolaita Sodo town, Ethiopia. Both descriptive and explanatory study designs were used and quantitative data were collected. A self-administered questionnaire was designed and distributed to randomly selected 403 (202 females & 201 males) unmarried youth. Data analysis was undertaken using SPSS software in which both descriptive and inferential statistical techniques were utilized for data presentation. The result showed that most (85.5%) of the young people without disabilities participated in the survey were not willing to have any type of personal relationships with persons with disabilities and the main reason for 44.2% of these respondents being the fear of reaction from family members. Furthermore, it was found that the level of willingness of youth without disabilities to engage in romantic love and marital relationships was not influenced by the socio-economic status of people with disabilities. Moreover, the result of binary logistic regression analysis showed that the willingness of respondents to have marital and romantic love relationship with persons with disabilities is significantly associated to the sex (OR = 2.376; *P* < 0.05; 95%CI = 1.210, 4.664), raised-up area (OR = 2.512; *P* < 0.01; 95%CI = 1.319, 4.783), age (OR = 2.886; P < 0.05; 95%CI = 1.012, 8.228) and the presence of person with disability in the family (OR = 3.945; *P* < 0.01; 95%CI = 1.648, 9.442) of respondents. The findings of the present research demonstrate that people with disabilities have continued to face stereotypes and discriminations. Such stereotypes extend to assuming them as asexual and unfit to carryout roles that arise from love or marital relationships which violates the rights of PWDs to form their own family and have children. It is therefore, important to raise the awareness of young people about the differences between disability and sexuality and that physical disability has nothing to do with sexuality and relationship formation.

## Introduction

It is hardly possible to put a straight forward definition of disability (Wasserman et al. 2016a, 2016b). The definitions as well as the extent to which people considered to be living with disability are included or excluded in major socio-economic activities vary from one culture to another (Eskay et al. 2012; Bunning et al. 2017). Young (2010) defines disability as the disadvantage or restriction of activity experienced by people living with impairments that result in exclusion and marginalization of the group from the mainstream social, economic, and political participations. According to WHO (1990), It is a restriction or inability (often due to denial of equal opportunity by the society) to perform activities by persons with certain levels of psychological, anatomical or physiological abnormalities in the way or within the range considered for a human being having psychological, physiological, or anatomical states that are labeled to be normal. Moreover, disability is physical or mental characteristic labeled or perceived as impairment or dysfunction and some personal or social limitation associated with that impairment (Wasserman et al. 2016a, 2016b). Since 1970s, there is a shift in the definition of disability from the medical model- associating disability with impairment- to a social model in which states that social and attitudinal barriers to inclusion are socially constructed phenomena and serve to create a disabling condition to people with some kinds of impairments (IFPA 2007).

People with disabilities tend to be disempowered and deprived of economic and social opportunities and security because of social and physical barriers in society (Wiman et al. 2002). The images of people with disabilities in both Eastern and Western cultures provide the bias for negative attitudes. Current assumptions combined with historical social portrayals of people with disabilities as “sick and suffering” make it difficult for these individuals to meet the standards of social norms and to be viewed independent of these images (Almaz, 2011). Moreover, persons with disabilities are the most marginalized groups when it comes to sexual and reproductive health issues (WHO 2009; Tanabe et al. 2015; Meza et al., 2017). In fact, many persons with disabilities enjoy the experience of marriage and family life. However, because of stigma and discrimination, lack of access to information and services, especially those on sexual and reproductive health, many do not marry and have children. According to Goodall et al. (2017), young people with functional disabilities are more likely to experience adverse employment, educational and relationship outcomes in the transition to adult life, with the greatest disadvantage experienced by females.

Persons with disabilities face various hindrances to their sexuality which includes lack of sexual self-esteem, failure to enjoy pleasurable sex, and failure to get sexual partners (Tepper 2000). The stereotype that ascribes asexuality to persons with disabilities is a general phenomenon in most societies. The stigma of asexuality will depend on the kind and extent of disability (Miller et al. (2009). It is not merely that the disabled body may not be aesthetically appealing according to social meanings of attractiveness, though that may be part of the reason persons with disabilities experience stigma. However, a major determining factor of the stigma is the extent to which the physical or mental disability has the potential to, or actually challenges the dominant norms governing sexuality. Experiencing a limiting long-term illness, impairment or significant health problem is associated with an increased likelihood for disabled adults of being single/unmarried and an increased likelihood of being divorced or separated: the potential implications of impairment for relationship status have additionally been shown to be different for men and women at different points in the life span (Clarke and Mckay 2008).

Women with disabilities are the most vulnerable and marginalized groups in today‘s society. The disability stereotyping compounded with gender-role dynamics has made women with disabilities the subject of double discrimination in many different facets of life (Eleni, 2016; Mostert 2016; Beleza 2003). Family prejudices reinforce the idea that girls with disabilities have neither sexual identity nor a right to find a partner. The fact that women with disabilities do not match the physical model promoted in the media inhibits recognition of their right to sexuality. Many men may find a sexual relationship with a woman with disabilities a difficult concept. This may be through ignorance or belief that it is taboo. Having a family of one’s own and having one’s entitlement to parenthood recognized can be the hardest things for a woman with a disability to achieve in comparison with a woman who is not disabled and even with a man with a disability (Council of Europe 2003). Women with disability in Ethiopia are vulnerable to discrimination, rape, beating, verbal abuse, and physical neglect (Berhanu, 2015; Spratt 2017).

Across the world, people with disabilities face attitudinal barriers including prejudice, stereotypes, and low expectations (Division for Social Policy and Development, 2016). Communities may believe that people with disabilities lack the necessary qualities to make successful marriage partners, and beliefs around disability being related to bad family spirits can lead to concerns that they will bring evil or misfortune with them if they marry into the family (Aley 2016). According to Haage (2017), the marital chances of people with disabilities are significantly smaller compared to their non-disabled counterparts. Many nondisabled people may doubt that people with disabilities can be fulfilling partners in any loving adult relationship (Wasserman et al. 2016a, 2016b). In spite of this, Abed et al. (2015) found no significant difference between handicapped and non-handicapped couples in compatibility and marital satisfaction.

In Ethiopia, the state of persons with disability in social situation can be explained by the nature of prevailing understanding of disability, in terms of the conceptualization of its causes, nature and consequences. As a matter of course, the birth of a child with disability has been recorded as source of shame, disagreement as well as divorce among some families. There is a general tendency to think of person with disability as weak, hopeless, dependent and unable to learn and the subject of charity (Eleni, 2016). In Ethiopia, people with disabilities often are not participants in society because of the overall belief that disabilities are a result of a curse and/or are punishments from a deity (Mesfin, 1999). Because of their inability to perform physical labor, individuals with disabilities are viewed as burdens to their immediate families for not being able to contribute to the family’s income (Almaz, 2011). In some parts of Ethiopia, large numbers of people also believe that disability is the result of contact with evil spirits or evil eye. The family of the leper person is also called a cursed family and no one of “able bodied” had interest to have marriage relationship with a family with a leper person (Beide, 2018).

A study by Etabezahu (2013) reveals that most people living with sensory disabilities in Ethiopia are sexually active and a quarter of them reported having multiple partners. The study found people with sensory disabilities are highly vulnerable to HIV/AIDS due to engagements in risky sexual practices mainly motivated by enhancing lower income. According to Belaynesh et al. (2017), relationships and motherhood proved a very rewarding option for women with disabilities in Ethiopia. Women with disability also expressed their need for intimacy regardless of society’s denial. Being involved in a relationship is, however, very difficult for disabled women. If they are in a relationship, the relationship may not continue because of reasons such as avoidance of men, financial problems to support a child in case of pregnancy, interference of siblings, and avoidance of disabled women.

People with disabilities in Ethiopia have, however, continued to face negative attitudes, stigma, and discrimination (Mesfin, 1999). A study by Almaz (2011) found that Ethiopian college students have negative attitude toward people living with disabilities. It was contended that people are deliberately choosing not to socially include and interact with people with disabilities, since the culture requires daily social and physical interactions. According to Eleni (2016), women with disability in Ethiopia who are never married face different challenges on their life. Unmarried women have less value for themselves; they believed that no one would want to marry disabled woman. Furthermore, they feared that the man might be mistreated by the society because of her when he was seen with her.

A previously undertaken study by Miller et al. (2009) in Texas, America, indicated that students were significantly more willing to have friendships and acquaintanceships with persons with mild to moderate disabilities and persons with sensory, health, and physical impairments. Students were least willing to marry or have a partnership with Persons with Disabilities, especially if the Persons with Disabilities had cognitive and psychiatric impairments. The finding was based on data collected from young Hispanic women preparing to work in humanities professions such as social work and rehabilitation counseling. It implies that the willingness of men to engage in personal relationships with disabled women was ignored in the study under consideration. Above all, the absence of adequate published empirical literature on the subject among other societies also made our knowledge very limited.

Understanding and recognizing that persons with disabilities are still exposed to and oppressed by prejudice and discrimination may be the first step in reducing prejudice (Marks 1997). Besides the fact that there are only few previously published studies in Ethiopia regarding the attitude of people living without disabilities towards those living with disabilities, most of the already available ones are institutional (e.g. Almaz, 2011; Eleni, 2016). In addition, other than revealing about the widely held stereotypes and negative attitudes towards persons living with disabilities, previous studies were not able to address the specific question about the willingness of persons without disabilities to engage in courtships with persons with disabilities. The purpose of the present research was, therefore, to assess the willingness of non-disabled youth to date and marry persons with disabilities in Wolaita Sodo town. In this context, this research aimed to answer the following basic research questions:

- Living in a social setting with long-held stereotypes about the sexuality of people living with disabilities, are non-disabled youth willing to form courtships with persons with disabilities?
- Are the socio-demographic characteristics of non-disabled persons (age, sex, religion, residential background, level of education, the presence/absence of previous relationship with disabled persons) associated to their willingness to date and marry persons with disabilities?
- Is the willingness of youth without disability to engage in love and marital relationships with persons with disabilities influenced by the Socio-Economic Status (Income, Education, and Employment, and occupation) of the later?

## Materials and methods

### Study design

The research involves both descriptive and explanatory study designs. A cross-sectional study which involves quantitative research approach was used in the present study. Quantitative research is the strategy that emphasizes quantification in the collection and analysis of data Bryman (2012). It seeks regularities in human lives, by separating the social world into empirical components called variables which can be represented numerically as frequencies or rate, whose associations with each other can be explored by statistical techniques, and accessed through researcher-introduced stimuli and systematic measurement (Payne and Payne 2004). The researchers used quantitative research approach mainly due to the reason that quantitative findings are likely to be generalized to a whole population or a sub-population because it involves the larger sample which is randomly selected (Carr 1994). Besides sampling, data analysis is less time consuming as it uses the statistical software such as SPSS (Connolly 2007).

### Method and source of data

First hand data were collected from research participants using survey research method. Given its advantage of enabling the researchers to undertake analysis of relationship between variables in addition to its generalizability, survey research method was preferred for this study. Moreover, survey method was chosen because of its inclusiveness in the types and number of variables that can be studied and requires minimal investment to develop and administer. A self-administered questionnaire was prepared, translated in to Amharic language (to ensure better understanding of the items), duplicated and was finally distributed to the survey respondents. A Pilot study was undertaken prior to the main process of data collection on similar population but different from the actual research samples in order to check issues related to the tools of data collection.

### Sample size and sampling technique

For the purpose of determining the sample size of survey participants, multi-stage stratified sampling technique was employed. In Wolaita Sodo town, there are seven administrative *kebeles* (the smallest governmental administrative units in Ethiopia). From these, three *kebeles*- *Wadu Amba*, *Fana Woniba* and *Arada Amba* were selected by using simple random sampling technique. Given that the statistics pertaining to the population size to each *kebele* were outdated and no recent data was available, the total population size in the study area was unknown. Hence, in order to determine the appropriate sample size; the researchers employed Cochran’s (1977) formula for calculating sample size of unknown population:

$$ n=\frac{{\mathrm{z}}^2\mathrm{pq}}{{\mathrm{e}}^2}=384 $$

Where, n is the sample size, z is the selected critical value of desired confidence level, p is the estimated proportion of an attribute that is present in the population, q = 1− p and e is the desired level of precision. In order to back-up the potential non-response rate, 5 % additional questionnaires were prepared in addition to the ones proportionate to the calculated sample size. Then, 403 questionnaires (384 + 19) were distributed to randomly selected young people in each *kebele* (202 females & 201 males).

The inclusion criteria to participate in the survey were: age, marital status, residence, ability to read and write the language used in the questionnaire, and a full consent to participate in the study. Accordingly, young people who were between 15 and 35 years of age, that are never married during the time of data collection, those who can read and write, that have a full consent to participate in the study, and those who are permanent residents of Wolaita Sodo town were included. On the other hand, people below 15 and above 35 years old, married, that are not permanent dwellers of the study area, did not have a full consent to participate in the survey, and those who cannot read and write were excluded from the survey.

### Instrument design

While some of the items in the questionnaire were taken from Miller et al. (2009) and contextualized to our research purpose, most of the items were constructed by the researchers depending on the specific research questions. The first part of the questionnaire contained socio-demographic variables including, age, sex, educational background, religion, and grownup area. Regarding age, a blank space was provided and respondents were asked to fill their appropriate age which is measured by the total number of years a person lived since birth. Sex was defined as biological differences and labeled with categories of “female” and “male”. In addition, educational background was categorized as “never attended school”, “1–8”, “9–12”, “college diploma”, “BA or BSc”, “MA or MSc & above”. Religion was categorized as “Orthodox Christian”, “Muslim”, “Protestant”, “Catholic”, “Jehovah”, “Adventist”, “Atheist”, “and “Other”. Moreover, grownup area was measured by whether the respondent was raised-up in “rural” or “urban” area.

The second section of the instrument contained questions aimed at examining respondents’ patterns of previous interaction with PWDs. The presence or absence of previous interaction with PWDs was measured by asking: “Have you had any regular interaction or experience of living with people with disabilities?” with response categories of “Yes” and “No”. The frequency of previous contact with PWDs was measured by asking “If yes, how often?” following the previous contingency question and with response categories of “daily”, “once a week”, “once a month”, “rarely”, “and “occasionally”. Furthermore, the presence of PWDs as family member was assessed by the question: “Do you have a family member (s) who has disability?” with response categories of “yes” and “no”. For those who answered “yes” to the previous question, the experience of interaction with PWDs was measured by asking: “how do you express your experiences in the relationship?” having responses of “unpleasant experience”, “pleasant experience”, and “indifferent”. Respondents’ willingness to engage in personal relationship with PWDs was assessed by the question: “Are you willing to engage in conjugal relationship (boy/girlfriend, spouse) with someone living with disability?” with response categories of “yes” and “no”. Finally, respondents’ potential reason for saying “no” to the same question was examined by a list of alternatives including: “fear of reaction from family members”, “fear of reaction from other members of the society”, “I don’t think s/he will be able to appropriately accomplish expected roles”, “s/he would be asexual”, “fear of probability of giving birth to disabled children”, “do not fulfill my criteria of beauty”, “reasons related to religion”, “no reason”, and “others”.

The third section contained Likert sacle items with the intention of measuring respondents’ attitude towards engaging in conjugal or romantic love relationships with PWDs. The section consisted of five positive and negative statements with a four scale Likert items. The questions include: “I feel less comfortable to be around a person with disability”, “I feel indifferent if I marry a person living with disability”, “I would rather prefer to remain unmarried than marrying someone with disability”, “I am ready to accept it as a fate of life in case I fall in love with someone living with disability”, and “I would never care about the disability status of a person when engaging in any type of relationship.” And the response categories for all the questions were: “4 = strongly agree”, “3 = agree”, “2 = disagree”, and “1 = strongly disagree”.

The fourth section of the questionnaire consisted of questions designed for the purpose of assessing the impacts of the socio-economic status (SES) of PWDs on the willingness of youth without disabilities to engage in conjugal relationships with PWDs. The objective of this section was to examine whether respondents’ level of willingness vary with varying socio-economic status of PWDs. This section, with similar items, was divided in to two sub-sections where the first deals with respondents’ willingness to engage in romantic love relationship with PWDs and the second one assesses their willingness to engage in marital relationship with PWDs. The socio-economic status of PWDs were differentiated as “educational status of PWDs” which ranged from “uneducated” to “PWDs having PhD”; “the employment status of PWDs” ranging from “unemployed” to “permanently employed”; “occupational status” ranging from “farmer” to “University professor”; and “income level” ranging from “with no income” to “15,000 birr^1^ & above”. The response categories for each of the above dimensions of socio-economic status and their respective categories were: “4 = very willing”, “3 = willing”, “2 = unwilling”, and “1 = very unwilling”.

### Method of data analysis

After the distributed questionnaires were returned, data completeness was checked. Accordingly, we found 359 cases that were appropriately and completely filled which make the response rate 93.5%. Then, the data were entered in to SPSS software and analyzed using both descriptive and inferential statistical techniques. Descriptive statistical techniques, including frequency distributions, percentage distributions, tables, and figures (charts) were used to analyze and present variables pertaining to the socio-demographic characteristics of respondents, the patterns of previous interaction with PWDs, willingness to engage in conjugal relationships with PWDs, and the possible reasons not to be willing to engage in such relationships with PWDs. In addition, mean, standard deviation, standard error, and variance were used to analyze respondents’ attitude towards engaging in conjugal relationships with PWDs. Above all, associations between the socio-demographic background of respondents (age, sex, religion, raised-up area, previous interaction with PWDs, the presence of PWDs in the family, and educational status) and their willingness to engage in conjugal relationships with PWDs was analyzed using coefficients of binary logistic regression. Considering respondents’ willingness as an outcome or dependent variable and the socio-demographic characteristics of respondents as independent variables, binary logistic regression analysis with Hosmer and Lemeshow test model was used. Independent variables having *P* value less than 0.05 were considered as significantly associated to the outcome variable. Values of Odd Ratios (OR) and confidence intervals were also considered in the analysis.

## Results

Table 1 presents results of the frequency and percentage distribution of respondents by their socio-demographic characteristics. It is shown that most (36.8%) of the research participants were within 25–29 age range, followed by respondents in 30–34 years old (23.7%). In addition, 11.1% of the respondents were between 15 and 19 years whereas 7.8% of them were 35 years old. Regarding the sex distribution of respondents, females constituted 22.8% of the respondents and the remaining 77.2% were males. Furthermore, data in the table also reveal that majority (39.8%) of the respondents were BA/Sc degree holders, 23.4% have completed 9–12 grades of education, 13.9% hold college diploma, 10% have graduated with Master’s degree & above, and 3.9% have never attended school.

**Table 1:** Socio-demographic characteristics of respondents

No.	Variables	Categories	Frequency (%)
1.	Age	15–19	40 (11.1%)
		20–24	74 (20.6%)
		25–29	132 (36.8%)
		30–34	85 (23.7%)
		35	28 (7.8%)
2.	Sex	Female	82 (22.8%)
		Male	277 (77.2%)
3.	Educational status	Never attended school	14 (3.9%)
		1–8	32 (8.9%)
		9–12	84 (23.4%)
		College diploma	50 (13.9%)
		BA/SC Degree	143 (39.8%)
		MA/SC Degree & above	36 (10%)
4.	Religion	Orthodox Christian	103 (28.7%)
		Muslim	23 (6.4%)
		Protestant	187 (52.1%)
		Catholic	25 (7%)
		Adventist (7th day)	15 (4.2%)
		Jova witness	4 (1.1%)
		Others	2 (0.6%)
5.	Raised-up area	Rural	104 (29%)
		Urban	255 (71%)
6.	Total		359 (100%)

As far as the religious affiliation of respondents is concerned, 52.1% were Protestants, 28.7% were Orthodox Christians, 7% were Catholics, 6.4% Muslims, 4.2% Adventists, 1.1% Jova witnesses, and the remaining (0.6%) were followers of other religions. Moreover, most (71%) of the research participants were raised-up in an urban area while 29% of them were raised-up in rural area.

Table 2 shows the frequency distribution of respondents in terms of whether or not they have had any previous regular interaction with PWDs, the type of interpersonal interaction maintained, the frequency of such interaction, including self-rated evaluation of the experiences respondents had in the relationship. The data show that 45.7% of them reported to have ever had regular social or interpersonal interaction with PWDs. From the total number of respondents who disclosed to have had an interaction with PWDs, majority of them reported to have infrequent type of interaction with PWDs (21.2% rarely, 11.1% occasionally) while 7.8% said to have interacted with PWDs on a daily basis.

**Table 2:** Patterns of previous interaction of respondents with PWDs

No.	Variables/questions	Categories	Frequency (%)
1.	Ever had any regular interaction with PWD	Yes	164 (45.7%)
		No	195 (54.3%)
2.	Frequency of previous interaction with PWD	Daily	28 (7.8%)
		Once a week	19 (5.3%)
		Once a month	13 (3.6%)
		Rarely	76 (21.2%)
		Occasionally	40 (11.1%)
		Others	6 (1.7%)
		Total	182 (50.7%)
		Missing	177 (49.3%)
3.	Ever had PWD as family member	Yes	33 (9.2%)
		No	326 (90.8%)
4.	Ever had personal relationship with PWD	Yes	12 (3.3%)
5.	Self-rated evaluation of experience in the relationship	No	347 (96.7%)
		Unpleasant	5 (1.4%)
		Pleasant	20 (5.6%)
		Indifferent	29 (8.1%)
		Total	54 (15%)
		Missing	305 (85%)
Total			359 (100%)

Regarding the type of previous interaction with PWDs, 9.2% of respondents who reported to have had previous interaction with PWDs, have had person/s with disabilities as a member of their family. In addition, 3.3% of them disclosed to have had personal relationship with PWDs. Furthermore, respondents’ self-rated evaluation of their experience during interaction with PWDs revealed that 8.1% felt indifferent, followed by 5.6% who reported to have a pleasant experience.

### Willingness of non-disabled youth to engage in love and marital relationship with PWDs

**Fig. 1:**
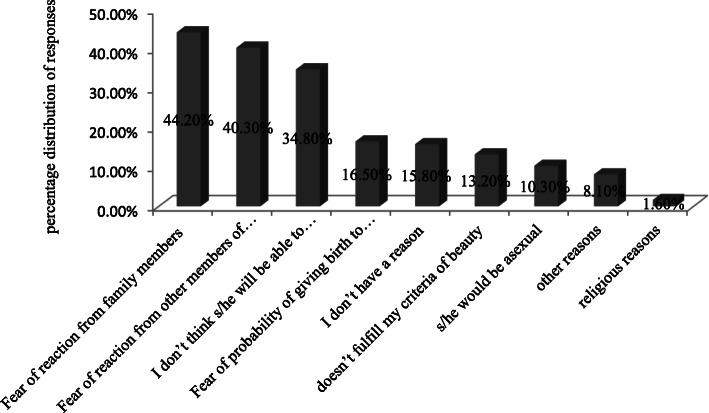
Respondents’ reasons for not being interested to have conjugal relationship with PWDs

Table 3 shows the frequency distribution of respondents in terms of their willingness to engage in conjugal relationships with PWDs. It is found that 85.5% of the respondents were not interested to engage in conjugal relationships with PWDs. The main reason for these respondents was reported to be the fear of potential reaction from family members (44.2%), followed by the fear of potential reaction from other members of the society (40.3%), an assumption that PWDs may not be able to carry out expected roles (34.8%), assumptions about the probability of giving birth to children with disability (16.5%), no reason (15.8%), PWDs do not fulfill the criteria of beauty (13.2%), belief that PWDs would be asexual (10.3%), and other reasons (8.1%) as shown in Fig. 1.

**Table 3:** Frequency distribution of respondents based on their willingness to engage in personal relationships with PWDs

No.	Question	Categories	Frequency (%)
1.	Are you interested to engage in Conjugal relationship with PWDs?	Yes	52 (14.5%)
		No	307 (85.5%)
	Total		359 (100%)

**Fig. 2:**
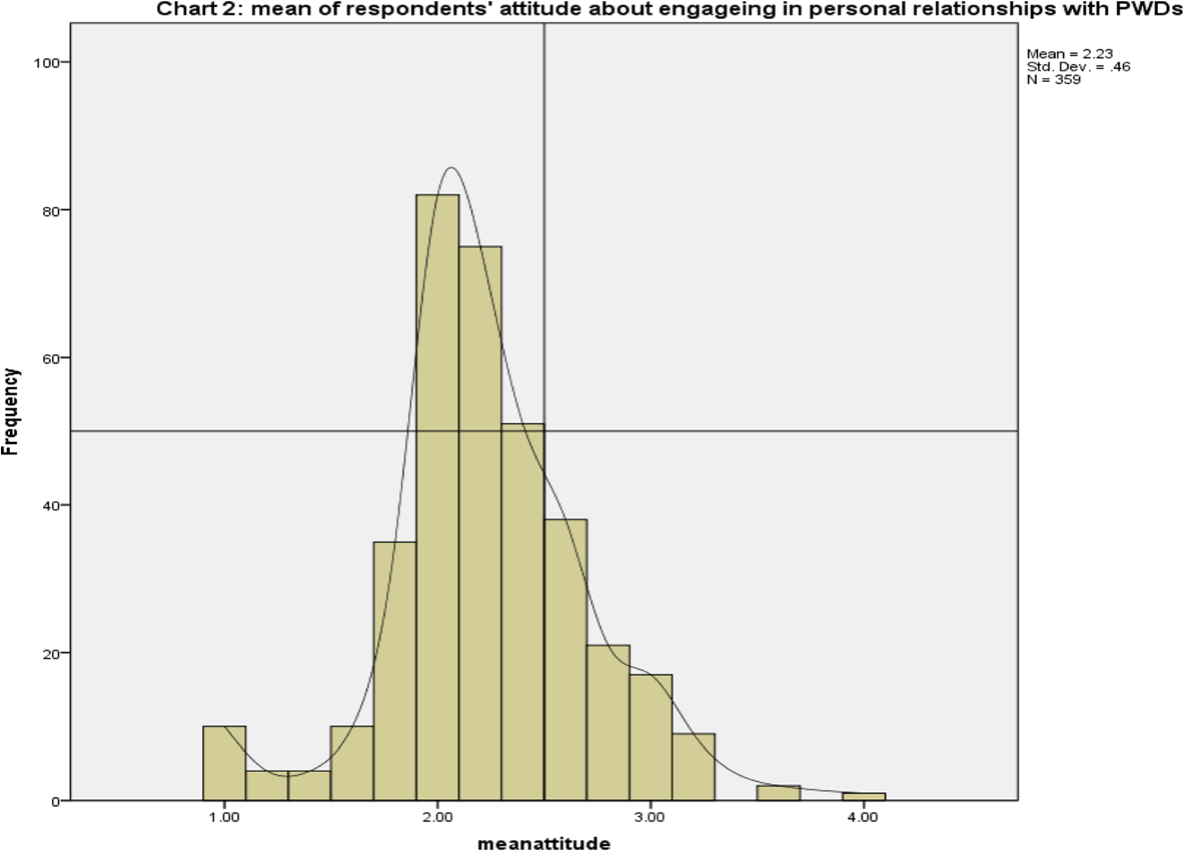
Mean of respondents’ attitude about engaging in personal relationships with PLWDs

Data in Table 4 and Fig. 2 present the frequency distribution of respondents in terms of attitude towards engaging in conjugal relationships with PWDs. Accordingly, it was found that most of the research participants did not have favorable attitude towards engaging in such relationships with PWDs. For instance, the mean for the statements: “I would rather prefer to remain unmarried than marrying someone with disability” (*M* = 1.9, *SD* = .79, *SE* = *.*042) and “I am ready to accept it as a fate of life in case I fall in love with someone with disability” (*M* = 2.3, *SD* = .74, *SE* = *.*039) indicate that respondents have negative attitude towards engaging in personal relationships with PWDs. The aggregate mean of respondents’ attitude was found to be 2.2 (*SD* = .46) and figure two shows that the attitude of most respondents lies below the average.

**Table 4:** Frequency distribution of respondents’ attitude towards engaging in conjugal relationships with PWDs

No.	Statements	***M***	***SE***	***SD***	Var.	Min	Max
1.	I feel less comfortable to be around a person with disability.	2.3	.051	.97	.94	1.0	4.00
2.	I feel indifferent if I marry a person living with disability	2.2	.039	.74	.55	1.0	4.00
3.	I would rather prefer to remain unmarried than marrying someone with disability.	1.9	.042	.79	.63	1.0	4.00
4.	I am ready to accept it as a fate of life in case I fall in love with someone with disability.	2.3	.039	.74	.55	1.0	4.00
5.	I would never care about the disability status of a person when engaging in any type of relationship.	2.4	.043	.81	.67	1.0	4.00

### Factors associated to willingness to engage in personal relationships with PWDs

Table 5 deals with the presentation of logistic regression coefficients using Hosmer and Lemenshow test model. It is found that sex (OR = 2.376; *P* < 0.05; 95%CI = 1.210, 4.664), raised-up area (OR = 2.512; *P* < 0.01; 95%CI = 1.319, 4.783), age (OR = 2.886; P < 0.05; 95%CI = 1.012, 8.228) and the presence of person with disability in the family (OR = 3.945; P < 0.01; 95%CI = 1.648, 9.442) of respondents are significantly associated to respondents’ willingness to engage in conjugal relationships with PWDs. The finding that OR = 2.376; 2.886; 2.512; and 3.945, respectively for sex, age, raised-up area, and having PWD in the family) indicates that the odds of willingness to engage in conjugal relationships with PWDs increases with every single change in both the sex, age, residential background (raised-up areas), and the presence of PWDs in the family of respondents. In other words, males, respondents that are older in age, respondents raised up in an urban area, and those having PWDs in their families are more likely (*P* < 0.05) to be willing to engage in conjugal relationships with PWDs than females, those younger in age, those raised up in rural areas, and respondents that do not have PWDs in their families.

**Table 5:** Binary logistic regression

Variables	B	S.E.	Wald	df	P Value	OR	95% C.I.	
Age	1.060	.535	3.932	1	.047	2.886*	(1.012,	8.228)
Sex	.865	.344	6.325	1	.012	2.376*	(1.210,	4.664)
Raised-up area	.921	.329	7.860	1	.005	2.512**	(1.319,	4.783)
Religion	.382	.681	.315	1	.575	1.465	(.386,	5.566)
Education	−.267	1.043	.065	1	.798	.766	(.099,	5.915)
Previous Interaction	.536	.330	2.632	1	.105	1.709	(.894,	3.265)
Having PWD in the family	1.372	.445	9.501	1	.002	3.945**	(1.648,	9.442)

**P < 0.01, *P < 0.05

### The socio-economic status of PWDs and willingness of youth without disabilities to engage in romantic love and marital relationships with PWDs

**Fig. 3:**
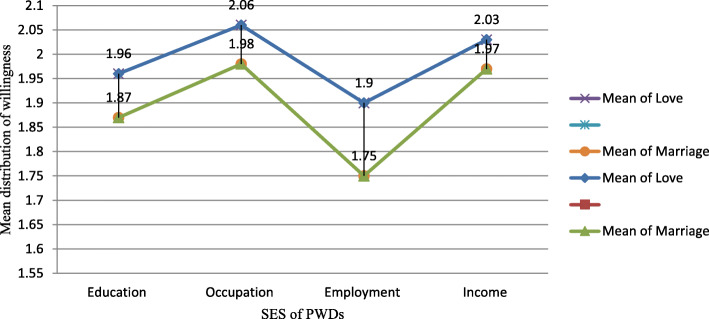
Mean distribution of willingness to engage in Love & Marital Relationships with PWDs

Data presented in Table 6 and Fig. 3 indicate that the socio-economic status (education, employment, income, and occupational status) of PWDs have no substantial impact on respondents’ willingness to engage both in romantic love and marital relationships with PWDs. As far as engaging in love relationship is concerned, respondents developed relatively favorable attitude to engage in romantic love relationship with PWDs having better occupational status (*M* = 2.06; *SD* = .64) while it was found that respondents are less influenced by the employment status of PWDs (*M* = 1.90; *SD* = .69). In case of engaging in marital relationship, respondents were more influenced by occupation (*M* = 1.98; *SD* = .68) and least influenced by employment status (*M* = 1.75; *SD* = .69) of PWDs. Therefore, while the occupational status of PWDs have a relatively highest influence, employment status have the least influence in case of engaging in both marital and romantic love relationships with PWDs. Above all, relatively speaking, respondents have shown stronger resistance to engage in marital relationship with PWDs than engaging in romantic love relationship, as shown in Fig. 3.

**Table 6:** Socio-economic status of PWDs & frequency distribution of Respondents’ willingness to engage in love & marital relationships with PWDs

Willingness to engage in Romantic Love relationship with PWDs						Willingness to engage in Marital relationship with PWDs			
No.	SES of PWDs	*M*	*SE*	*SD*	Var	*M*	*SE*	*SD*	Var
1.	Education	1.96	.034	.65	.426	1.87	.033	.63	.401
2.	Occupation	2.06	.034	.64	.419	1.98	.036	.68	.472
3.	Employment	1.90	.036	.69	.482	1.75	.036	.69	.477
4.	Income	2.03	.035	.67	.462	1.97	.036	.69	.483

## Discussion

People with physical disabilities (PWPD) are negatively stereotyped by most of society. Those PWD are then ostracized into having no self-esteem or worth in various social situations (Santuzzi 2011). They tend to be disempowered and deprived of economic and social opportunities and security because of social and physical barriers in society (Wiman et al. 2002). Moreover, persons with disabilities are the most marginalized groups when it comes to sexual and reproductive health issues (WHO 2009). Persons with disabilities may be perceived as of less value as a wife and as a husband, and their sexuality goes unrecognized. Above all, the “marketability” of young women with disabilities as a spouse is compensated for by the family by ensuring that they are employable and economically independent (Addlakha 2007). A study by Etabezahu (2013) reveals that most people living with sensory disabilities in Ethiopia are sexually active and a quarter of them reported having multiple partners. The present research aimed at assessing the willingness of young people without disability to engage in conjugal relationships with PWDs in Wolaita Sodo town. A self-administered questionnaire was distributed to randomly selected 403 never married young people between 15 and 35 years of age. Quantitative data were collected, entered in to a statistical package for social sciences software, and analyzed using both descriptive and inferential statistical techniques.

It was found that most of the young people participated in the present research (85.5%) reported to be unwilling to engage in any form of personal relationships with PWDs and the main reason for most (44.2%) of these segments of the respondents was the fear of reaction from family members. The finding of the present study is consistent with Miller et al. (2009). The authors found that students they studied were least willing to marry or have a partnership with Persons with Disabilities, especially if the Persons with Disabilities had cognitive and psychiatric impairments. A study by Almaz (2011) found that Ethiopian college students have negative attitude toward people living with disabilities. It was contended that people are deliberately choosing not to socially include and interact with people with disabilities, since the culture requires daily social and physical interactions. Despite constituting over 10% of the world’s population, persons with disabilities are often marginalized and their needs are overlooked or neglected. They have often been denied the right to establish relationships and to decide whether, when, and with whom to have a family. Many have been subjected to forced sterilizations, forced abortions, or forced marriages (WHO 2009). According to Eleni (2016), Women with disability in Ethiopia who are never married face different challenges on their life. Unmarried women have less value for themselves; they believed that no one would want to marry disabled woman. Furthermore, they feared that the man might be mistreated by the society because of her when he was seen with her.

The results of Likert scale analysis in the present study also found that most of the research participants do not have favorable attitude towards engaging in conjugal relationships with PWDs. The aggregate mean of respondents’ attitude was found to be 2.2 (*SD* = .46) and figure two showed that the attitude of most respondents lies below the average. The finding of the present study, however, contradicts to Staniland (2009) who found that attitudes towards disabled people have improved, on the whole; people are less likely to think of disabled people as getting in the way or with discomfort and awkwardness. Conversely, they are more likely to think of disabled people as the same as everybody else. The contradiction in the findings of the two studies might have resulted from differences in the socio-economic and cultural contexts in which the researches were undertaken. Antonak and Livneh (2000) pointed out that the investigation of attitudes towards persons with disabilities requires innovative experimental methods and psychometrically sound instruments that are reliable, valid, and multidimensional. Without such instruments, it will not be possible to obtain conclusive answers to important research questions concerning the relationship between these attitudes and the acceptance and integration of persons with disabilities into society.

In an attempt to find out the association between socio-demographic factors (age, sex, religion, raised-up area, education, and previous interaction with PWDs) of respondents and their willingness to engage in conjugal relationships with PWDs, a binary logistic regression analysis was undertaken. Consequently, the result has shown that sex (OR = 2.376; *P* < 0.05; CI = 1.210, 4.664), raised-up area (OR = 2.512; *P* < 0.01; CI = 1.319, 4.783), age (OR = 2.886; P < 0.05; CI = 1.012, 8.228) and the presence of person with disability in the family (OR = 3.945; P < 0.01; CI = 1.648, 9.442) of respondents are significantly associated to respondents’ willingness to engage in conjugal relationships with PWDs. Nevertheless, no considerable association has been found between PWDs’ socio-economic status (education, income, occupation, and employment status) and willingness to have marital or romantic love relationships with PWDs. The finding contradicts to DSPD’s (2016 cited in Rohwerder 2018) conclusion that socio-economic issues can affect attitudes towards disability where poorer people with disabilities may face more stigma, stereotype and discrimination than the more economically advantaged people with disabilities.

Miller et al. (2009) found a statistically significant interaction between category of disability, type of disability, level of severity of disability and willingness to have personal relationship with PWD. In addition, they pointed out that students reported a willingness to have a friendship or acquaintanceship with a PWD even if the disabilities were severe, yet they were essentially unwilling to date or marry anyone with a disability. Bond Disability and Development group (DDG) (2017) also found a relationship between severity and type of disability and level of stigma faced by PWDs: People with intellectual disabilities and people with severe mental health problems are generally more stigmatized than people with physical or sensory disabilities. According to Goodall et al. (2017), young people with functional disabilities are more likely to experience adverse employment, educational and relationship outcomes in the transition to adult life, with the greatest disadvantage experienced by females. Experiencing a limiting long-term illness, impairment or significant health problem is associated with an increased likelihood for disabled adults of being single/unmarried and an increased likelihood of being divorced or separated: the potential implications of impairment for relationship status have additionally been shown to be different for men and women at different points in the life span (Clarke and Mckay 2008). According to Belaynesh, et al. (2017), relationships and motherhood proved a very rewarding option for women with disabilities. Women with disability also expressed their need for intimacy regardless of society’s denial.

## Conclusion

Results of the present study revealed that most young people without disabilities are not willing to have conjugal relationships with people with disabilities in the study area. Such strong resistance to establish marital and romantic love relationships with PWDs were witnessed even under circumstances in which the socio-economic status of PWDs is relatively better, i.e. PWDs are better educated, have better employment and occupational status, and are high income earners. It was also revealed that differences in the level of willingness to engage in conjugal relationships with PWDs are partly the result of youth’s differences in sex, age, the presence of PWDs in the family, and raised-up area of respondents where males, older in age, have PWDs in the family, and those raised-up in an urban area are more willing to have such relationships than their rural, younger aged, do not have PWDs in the family, and female counterparts. The findings of both the present research and most other related previous studies demonstrate that people with various types of disabilities have continued to face stereotypes and discriminations. Such stereotypes extend to assuming them as asexual and unfit to carryout roles that arise from love or marital relationships which violates the rights of PWDs to form their own family and have children. It is therefore, important to raise the awareness of young people about the differences between disability and sexuality and that physical disability has nothing to do with sexuality and relationship formation.

## Data Availability

The data used to support the findings of this study are available from the corresponding author upon reasonable request.

## Abbreviations

PWDs: Persons with disabilities
UNFPA: United Nations Population Fund
SPSS: Statistical package for social sciences
WHO: World Health Organization
HIV/AIDS: Human immune virus/acquired immune deficiency syndrome

## Footnotes

1 1 US Dollar = 36 Ethiopian birr during the period of the study
